# A Mendelian randomization study on the effects of plasma lipids on irritable bowel syndrome and functional dyspepsia

**DOI:** 10.1038/s41598-023-50459-9

**Published:** 2024-01-02

**Authors:** Mengmeng Xu, Deliang Liu, Yuyong Tan, Jian He, Bingyi Zhou

**Affiliations:** 1 Department of Gastroenterology, The Second Xiangya Hospital, Central South University, No.139 Renmin Middle Road, Changsha, 410007 China; 2 Research Center of Digestive Disease, Central South University, Changsha, China; 3 Department of Gastroenterology, Nanfang Hospital, Southern Medical University, Guangzhou, 510515 China

**Keywords:** Genetics, Gastroenterology, Health care

## Abstract

Although functional gastrointestinal disorder (FGID) is a common clinical condition, its risk factors remain unclear. We performed a Mendelian randomization study to explore the association between plasma lipids and the risk of FGID. Instrumental variables closely related to six plasma lipids were obtained from the corresponding genome-wide association studies, and summary-level data on FGID, including irritable bowel syndrome (IBS) and functional dyspepsia (FD), were extracted from the FinnGen study. The primary inverse variance weighted method and other supplementary analyses were used to evaluate the causal relationship between diverse plasma lipids and FGID. For each increase in the standard deviation of triglyceride levels, there was a 12.0% increase in the risk of IBS rather than that of FD. Low- and high-density lipoprotein cholesterol, total cholesterol, apolipoprotein A, and apolipoprotein B levels were not associated with the risk of IBS or FD. Through this study, we identified the causal role of triglycerides in the pathogenesis of IBS, which could benefit further basic and clinical research.

## Introduction

Functional gastrointestinal disorders (FGIDs), now termed disorders of gut–brain interaction^1^, are prevalent as they affect more than one-third of the population^2^, and refer to symptoms such as abdominal discomfort, diarrhea, constipation, bloating, fullness, nausea, and vomiting without underlying structural defects^3^. Irritable bowel syndrome (IBS) and functional dyspepsia (FD) are the two most common types of the 26-adult-classified FGIDs identified using the Rome criteria. However, the pathogenesis of these diseases remains unclear^4^. The possible pathological mechanisms of FGID, including gut–brain dysfunction, psychological factors, chronic infections, disorders of the intestinal microbiota, systemic immune activation, alterations in intestinal permeability, low-grade mucosal inflammation, abnormalities in the bile salt metabolism, abnormalities in the serotonin metabolism, and genetic factors, have been widely studied currently^5^. However, most of the present studies are cross-sectional or case–control observational studies, in which potential residual confounding factors and reverse causality issues could affect the results.

Several studies on hormones, blood lipids, and proteins in patients with FGIDs have been conducted because its diagnosis is based on symptom criteria that lack biochemical markers^6–9^. Some researchers have found that the concentration of plasma lipids, especially triglycerides, is significantly higher in patients with IBS than that in controls^6,10^, and that lipid intermediates or end products of cholesterol metabolism are more prominent in IBS than in FD^8^. Some researchers believe that the triglyceride levels increase due to the pathophysiological changes such as the low-grade mucosal inflammation which caused by IBS, increased intestinal mucosal permeability, and abnormal intestinal motility associated with IBS would reduce the absorption of fats in the gastrointestinal tract^10^. Further, evidence shows that one of the main characteristics of patients with IBS is poly-unsaturated fatty acid malabsorption^11^. In contrast, some studies have suggested that hypertriglyceridemia is a risk factor rather than the result of IBS, as the use of lipid-lowering drugs can reduce the clinical symptoms in patients with IBS^12,13^. These two contradictory views make it difficult to determine a causal relationship between elevated triglyceride levels and IBS in traditional observational studies. Simultaneously, it is not easy to study the results by conducting randomized controlled trials for the high costs, long experiment time and ethical concerns.

Mendelian randomization (MR) is an ideal epidemiological approach that uses genetic instrumental variables (IVs) in non-experimental data to assess the causal effect of exposure on outcomes to enhance a causal inference^14^. Genetic variants are relatively independent of self-selected behaviors and are established long before disease manifestation, as they are randomly assigned to offspring. This is equivalent to the random process in randomized controlled clinical trials. Therefore, the MR method can be used in cases in which the exposure factors are expensive or difficult to assess, to eliminate residual confounding factors, and prevent reverse causality. Considering the unclear causal relationship between increased plasma lipid levels and FGID, in this study, we conducted a MR study to investigate the association between plasma lipids and the risk of FGID.

## Materials and methods

### Data source and open genome-wide association studies (GWAS) statistics

Data related to elevated levels of plasma lipids were obtained from the UK Biobank (UKB), a repository of biomedical data and research resources collected the genetic and clinical information on half a million participants in the United Kingdom^15^. The consortium of FGID, which includes IBS and FD, belongs to the FinnGen study^16^, a new study that integrates genetic information with digital healthcare data from people in Finland.

### Single nucleotide polymorphism (SNP) selection and assumption

**Figure 1:**
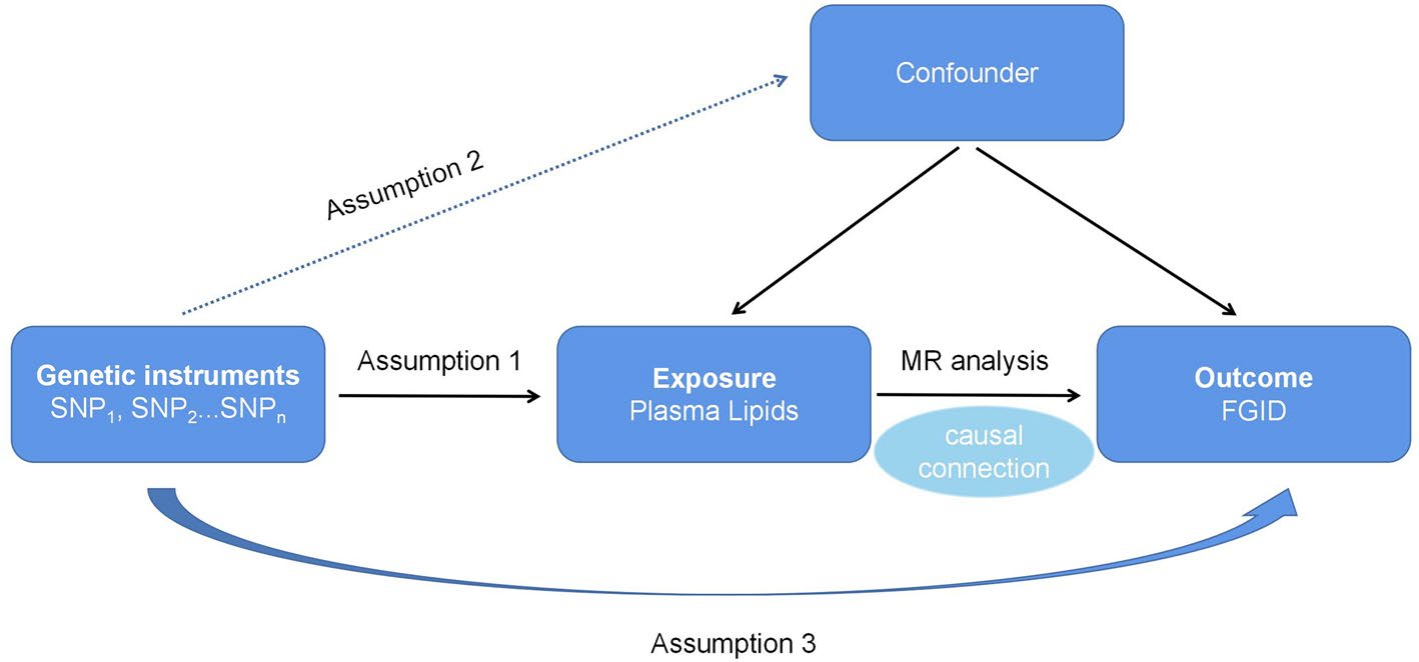
Flow diagram of the Mendelian randomization (MR) study. (1) The IVs are strongly associated with plasma lipids; (2) The genetic IVs do not affect the outcome through the confounders; (3) The genetic IVs do not affect functional gastrointestinal disease (FGID) directly, only via indirect exposure.

The assumptions used for the genetic variation met the following three basic requirements: (1) this variation was strongly associated with exposure (related hypothesis); (2) the variance did not affect the outcome because of the confounding factors (independence hypothesis); and (3) this variant did not directly affect the through indirect exposure (excluding hypothesis) (Fig. 1). For SNP selection, we set the statistical significance level (p < 5 × 10^–8^) to rigorously confirm genome-wide significant correlations to satisfy our hypothesis (1). In order to attenuate the disequilibrium of the linkage in SNP, we established the threshold (R^2^ < 0.001) and a specific mutation frequency, the minor allele frequency (MAF > 1%). For hypotheses (2) and (3), we examined each SNP in PhenoScanner, a public database on the association between human genetics and phenotypes, and excluded genome-wide SNPs that were significantly associated with potential confounders. Palindromic variants were removed to verify the harmonization of the variants, as it was challenging to verify the alleles for which they were correctly directed.

### Statistical analysis of primary MR

We chose the inverse-variance weighted (IVW) method, which is considered the most effective analysis method using reliable IVs, as the main analysis technique in our study^17^. When the pleiotropic effects of IVs were absent, and the sample size was large enough, the IVW estimate was reliable, accurate, and close to the true value^18^. When heterogeneity was statistically significant (p < 0.05), the multiplicative random effects IVW model was used. Otherwise, a fixed effects model was used. In addition to IVW, other reliable techniques were used to guarantee the accuracy and consistency of the results, including the MR-Egger method^19^, weighted median^18^, penalized weighted median estimator^20,21^, and maximum likelihood method^22^. Using scatter, funnel plots and forest to visualize the results and demonstrate the validity and stability of MR study.

The heterogeneity test was used to determine the conformance of each SNP using the MR-Egger and inverse-variance weighted techniques to calculate Cochran’s Q statistics. Heterogeneity was considered statistically significant at p < 0.05. A leave-one-out analysis was performed, and the results were compared with those of a global IVW analysis to confirm the reliability and stability of the causal relationship^14^. The MR-Egger intercept test was used to assess horizontal pleiotropy^14^. All analyses were performed by the R software (version 4.1.1) and the two-sample MR package.

## Results

### GWASs of plasma lipids levels

The GWAS of various plasma lipids levels were obtained from the UK Biobank, covering 187,365–403,943 participants, and 125–362 highly associated SNPs were recovered. The FinnGen study, which included 4605 and 4376 participants, produced the GWAS for IBS and FD, respectively (Table 1). Robust genetic IVs for six kinds of plasma lipidi levels were identified by the FinnGen database using open GWAS platforms (https://gwas.mrcieu.ac.uk) to determine the effect of plasma lipids levels-related genetic IVs on IBS and FD. The functions of “extract_outcome_data” and “harmonise_data” was used in conducting the identification process, and 81–315 SNPs were extracted for the subsequent causality analysis (Tables 2, 3).

**Table 1:** The list of genome-wide summary association studies (GWAS) used in the study.

Exposures	Consortium	GWAS ID	Sample size	Number of SNPs	No.of strongly related SNPs	Adjustment	Population
LDL-cholesterol	UK biobank	ieu-b-110	403,943	12,321,875	177	Sex, age and the first ten genetic principle components	European
HDL-cholesterol	UK biobank	ieu-b-109	403,943	12,321,875	362		European
Total cholesterol	UK biobank	ieu-a-301	187,365	2,446,982	125		European
**Triglycerides**	**UK biobank**	**ieu-b-111**	**403,943**	**12,321,875**	**312**		**European**
Apolipoprotein A1	UK biobank	ieu-b-107	403,943	12,321,875	299		European
Apolipoprotein B	UK biobank	ieu-b-108	403,943	12,321,875	198		European

Outcomes	Consortium	GWAS ID	No. of cases	Control		Adjustment	Population
Irritable bowel syndrome	FinnGen	finn-b-K11_IBS	4605	182,423		Sex, age and the first ten genetic principle components	European
Functional dyspepsia	FinnGen	finn-b-K11_FUNCDYSP	4376	189,695			European

Significant values are in bold.

**Table 2:** MR analysis results of different plasma lipids and irritable bowel syndrome.

Plasma lipids	MR method	No. SNP	β	SE	OR (95% CI)	p
LDL-cholesterol	MR Egger	155	0.201	0.089	1.223 (1.027, 1.456)	0.025
	Weighted median		0.175	0.097	1.191 (0.984, 1.440)	0.072
	IVW (fixed effects)		0.090	0.061	1.094 (0.971, 1.232)	0.140
	Maximum likelihood		0.090	0.061	1.095 (0.971, 1.234)	0.139
	Penalised weighted median		0.176	0.099	1.192 (0.982, 1.447)	0.075
HDL-cholesterol	MR Egger	315	− 0.106	0.085	0.900 (0.762, 1.062)	0.211
	Weighted median		− 0.129	0.094	0.879 (0.732, 1.056)	0.168
	IVW (random effects)		− 0.030	0.056	0.970 (0.870, 1.083)	0.593
	Maximum likelihood		− 0.030	0.051	0.970 (0.878, 1.073)	0.556
	Penalised weighted median		− 0.130	0.090	0.878 (0.736, 1.047)	0.148
Total cholesterol	MR Egger	118	0.148	0.084	1.159 (0.983, 1.368)	0.084
	Weighted median		0.138	0.081	1.148 (0.980, 1.345)	0.088
	IVW (fixed effects)		0.044	0.052	1.045 (0.942, 1.158)	0.406
	Maximum likelihood		0.044	0.053	1.045 (0.943, 1.159)	0.403
	Penalised weighted median		0.139	0.083	1.149 (0.976, 1.353)	0.094
Triglycerides	MR Egger	276	0.101	0.079	1.107 (0.948, 1.293)	0.201
	Weighted median		0.240	0.091	1.272 (1.064, 1.520)	0.008
	**IVW (fixed effects)**		**0.114**	**0.053**	**1.120 (1.010, 1.243)**	**0.032**
	Maximum likelihood		0.115	0.053	1.122 (1.011, 1.245)	0.030
	Penalised weighted median		0.242	0.093	1.274 (1.062, 1.528)	0.009
Apolipoprotein A1	MR Egger	261	− 0.037	0.093	0.964 (0.804, 1.156)	0.693
	Weighted median		0.026	0.095	1.026 (0.852, 1.235)	0.786
	IVW (fixed effects)		− 0.050	0.055	0.951 (0.853, 1.059)	0.361
	Maximum likelihood		− 0.051	0.055	0.951 (0.853, 1.060)	0.360
	Penalised weighted median		0.029	0.094	1.029 (0.856, 1.237)	0.760
Apolipoprotein B	MR Egger	179	0.134	0.071	1.143 (0.995, 1.313)	0.060
	Weighted median		0.142	0.090	1.152 (0.966, 1.374)	0.114
	IVW (fixed effects)		0.091	0.053	1.095 (0.988, 1.214)	0.083
	Maximum likelihood		0.092	0.053	1.096 (0.989, 1.216)	0.081
	Penalised weighted median		0.142	0.082	1.153 (0.982, 1.353)	0.081

*No. SNP* number of SNPs included in the analysis, *β* the regression coefficient based on plasma lipids raising effect allele, *SE* standard error.

p < 0.05 represents the causal link of plasma lipids with IBS.

Significant values are in bold.

**Table 3:** MR analysis results of different plasma lipids and functional dyspepsia.

Plasma lipids	MR method	No. SNP	β	SE	OR (95% CI)	p
LDL-cholesterol	MR Egger	155	0.083	0.091	1.086 (0.909, 1.298)	0.366
	Weighted median		0.117	0.099	1.124 (0.926, 1.364)	0.236
	IVW (fixed effects)		0.044	0.062	1.045 (0.926, 1.180)	0.479
	Maximum likelihood		0.045	0.062	1.046 (0.926, 1.181)	0.472
	Penalised weighted median		0.122	0.100	1.129 (0.929, 1.373)	0.222
HDL-cholesterol	MR Egger	315	− 0.007	0.079	0.993 (0.850, 1.160)	0.932
	Weighted median		− 0.073	0.088	0.930 (0.783, 1.105)	0.408
	IVW (fixed effects)		− 0.010	0.052	0.990 (0.893, 1.097)	0.843
	Maximum likelihood		− 0.010	0.052	0.990 (0.893, 1.096)	0.842
	Penalised weighted median		− 0.073	0.090	0.930 (0.780, 1.109)	0.420
Total cholesterol	MR Egger	81	0.107	0.092	1.113 (0.930, 1.333)	0.246
	Weighted median		0.123	0.082	1.131 (0.964, 1.328)	0.131
	IVW (fixed effects)		0.062	0.053	1.064 (0.958, 1.181)	0.248
	Maximum likelihood		0.062	0.054	1.064 (0.958, 1.182)	0.246
	Penalised weighted median		0.124	0.083	1.132 (0.962, 1.331)	0.134
Triglycerides	MR Egger	276	0.016	0.084	1.016 (0.862, 1.198)	0.848
	Weighted median		− 0.023	0.091	0.977 (0.818, 1.167)	0.800
	IVW (fixed effects)		0.050	0.054	1.051 (0.946, 1.168)	0.351
	Maximum likelihood		0.051	0.054	1.052 (0.946, 1.169)	0.348
	Penalised weighted median		− 0.022	0.087	1.978 (0.824, 1.161)	0.799
Apolipoprotein A1	MR Egger	261	0.068	0.091	1.070 (0.896, 1.278)	0.454
	Weighted median		0.022	0.095	1.023 (0.849, 1.232)	0.813
	IVW (fixed effects)		0.005	0.057	1.005 (0.901, 1.122)	0.924
	Maximum likelihood		0.005	0.056	1.005 (0.900, 1.123)	0.924
	Penalised weighted median		0.041	0.094	1.042 (0.867, 1.252)	0.662
Apolipoprotein B	MR Egger	179	− 0.013	0.075	0.987 (0.852, 1.144)	0.867
	Weighted median		− 0.045	0.092	0.956 (0.799, 1.144)	0.622
	IVW (fixed effects)		0.006	0.054	1.006 (0.906, 1.118)	0.905
	Maximum likelihood		0.006	0.054	1.006 (0.906, 1.118)	0.905
	Penalised weighted median		− 0.046	0.089	0.955 (0.802, 1.138)	0.606

*No. SNP* number of SNPs included in the analysis, *β* the regression coefficient based on plasma lipids raising effect allele, *SE* standard error.

p < 0.05 represents the causal link of plasma lipids with FD.

### Primary MR analysis

**Figure 2:**
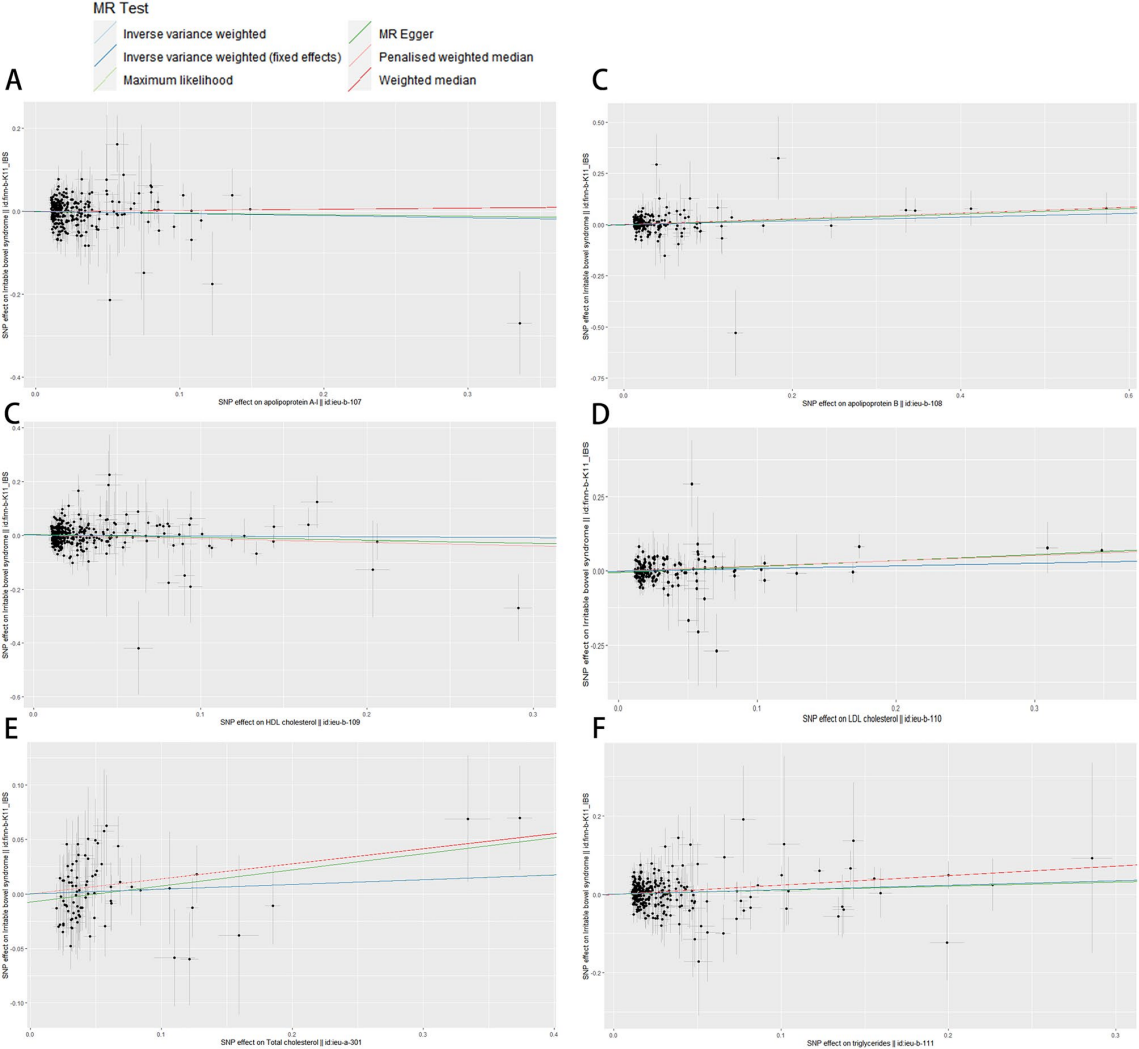
Forest plot showing associations of genetically predicted plasma lipids with risk of irritable bowel syndrome (IBS). *CI* confidence interval, *OR* odds ratio. (**A**) Analysis of apolipoprotein A1 and IBS. (**B**) Analysis of apolipoprotein B and IBS. (**C**) Analysis of HDL cholesterol and IBS. (**D**) Analysis of LDL cholesterol and IBS. (**E**) Analysis of total cholesterol and IBS. (**F**) Analysis of triglycerides and IBS.

**Figure 3:**
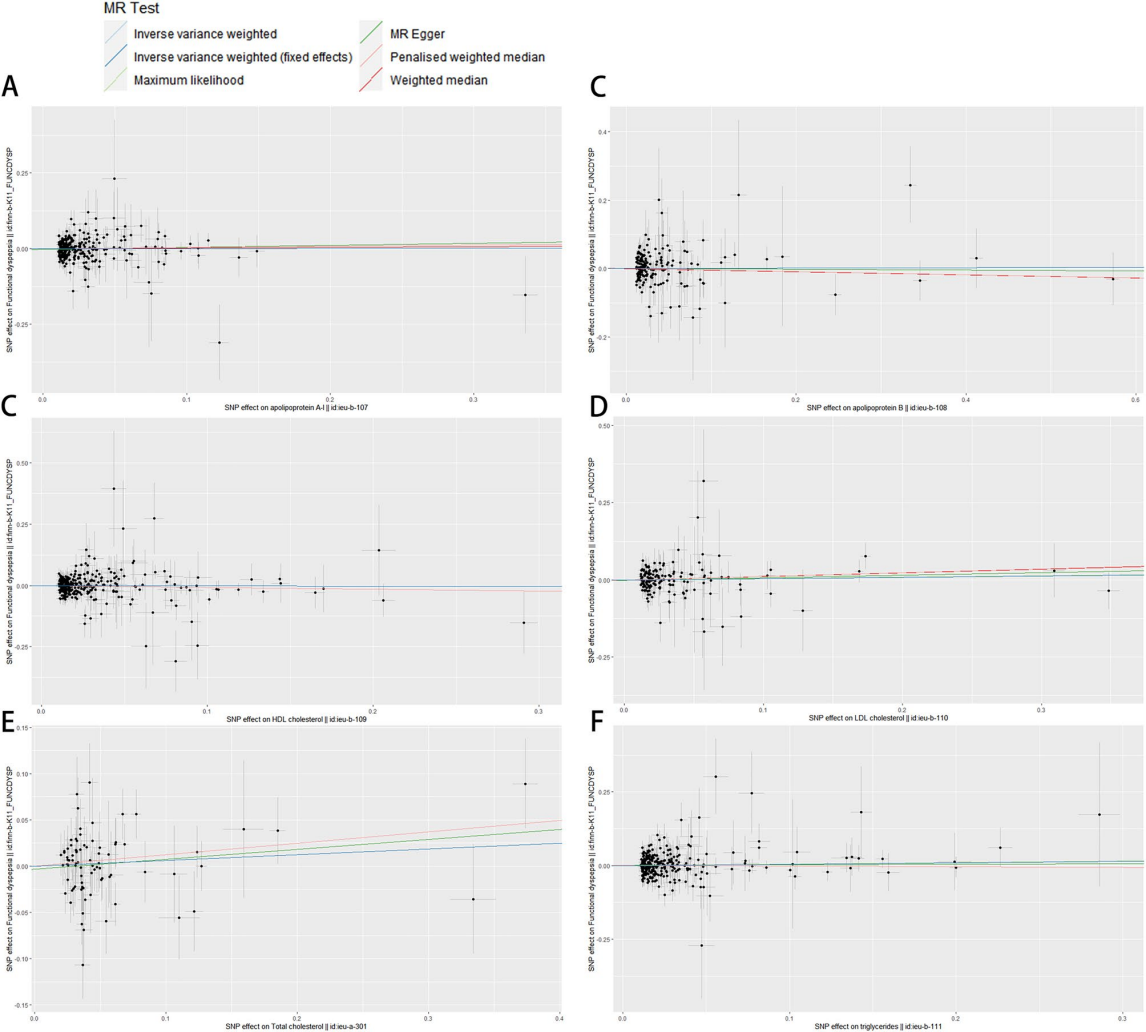
Forest plot showing associations of genetically predicted plasma lipids with risk of functional dyspepsia (FD). *CI* confidence interval, *OR* odds ratio. (**A**) Analysis of apolipoprotein A1 and FD. (**B**) Analysis of apolipoprotein B and FD. (**C**) Analysis of HDL cholesterol and FD. (**D**) Analysis of LDL cholesterol and FD. (**E**) Analysis of total cholesterol and FD. (**F**) Analysis of triglycerides and FD.

No causal relationship was found between the five plasma lipids (low-density lipoprotein (LDL)-cholesterol, high-density lipoprotein (HDL)-cholesterol, total cholesterol, apolipoprotein A1, and apolipoprotein B) and FGID (including IBS and FD) (Tables 2, 3). Moreover, the IVW method did not reveal a causal relationship between triglycerides and FD. However, it revealed a positive causal relationship between triglycerides and IBS (IVW, OR/95% CI 1.120/[1.010, 1.243], p < 0.05) (Table 2). The risk of IBS was increased by 12.0% for each standard deviation increase in genetically determined plasma lipid levels. Moreover, we have not only used the primary IVW analysis method but also other statistical methods to verify the accuracy of the main results, such as weighted median estimator, the MR-Egger, maximum likelihood estimation, and penalized weighted median estimator methods. Visualizing the effect size of each MR method by using a scatter plot (Figs. 2, 3); visualizing the individual SNP estimates of the outcomes by using a forest plot (Supplementary Figs. 1, 2); and the distribution balance of the single SNP effects is showed by using a funnel plot (Supplementary Figs. 3, 4). These plots suggest that the effects of each SNP and its distribution were in equilibrium.

### Sensitivity analysis

Using the Cochran’s Q test to measure heterogeneity. The effects of HDL-cholesterol on IBS were significantly and statistically heterogeneous among the genetic IVs (IVW, p = 0.01) (Table 4). The IVW multiplicative random-effects model was applied to the associations to calculate causal effects. Furthermore, no significant statistical heterogeneity was found among the genetic IVs for the effects of the remaining five plasma lipids on IBS and the effects of all six plasma lipids on FD (IVW, p ≥ 0.05). Accordingly, the fixed-effects IVW model was performed in the primary MR analysis.

**Table 4:** The heterogeneity test of plasma lipids genetic variants in IBS and FD Genome-wide summary association study (GWAS) datasets.

Traits (outcome)	Plasma lipids (exposure)	Methods	Q	Q-dif	Q-pval
IBS	LDL-cholesterol	MR Egger	140.705	153	0.753
		IVW	143.654	154	0.714
	HDL-cholesterol	MR Egger	378.956	313	0.006
		IVW	380.693	314	0.005
	Total cholesterol	MR Egger	69.435	79	0.820
		IVW	69.918	80	0.782
	Triglycerides	MR Egger	281.715	274	0.361
		IVW	281.760	275	0.377
	Apolipoprotein A1	MR Egger	284.656	259	0.131
		IVW	284.696	260	0.140
	Apolipoprotein B	MR Egger	161.486	177	0.792
		IVW	162.306	178	0.794
FD	LDL-cholesterol	MR Egger	154.830	153	0.443
		IVW	155.173	154	0.458
	HDL-cholesterol	MR Egger	322.847	313	0.339
		IVW	322.851	314	0.353
	Total cholesterol	MR Egger	90.274	79	0.181
		IVW	90.731	80	0.193
	Triglycerides	MR Egger	306.203	274	0.088
		IVW	306.540	275	0.093
	Apolipoprotein A1	MR Egger	263.582	259	0.409
		IVW	264.378	260	0.413
	Apolipoprotein B	MR Egger	193.386	177	0.189
		IVW	193.541	178	0.202

Leave-one-out analysis was used to assess the effects of a single SNP on the final MR results. After the sequential omission of the single SNP, the other causal effects of various plasma lipids on FGID found in the leave-one-out analysis were consistent with those found in the primary MR studies, showed that no single SNP significantly affected the final result (Supplementary Figs. 5, 6). This suggests that these MR studies are credible and reliable.

A horizontal pleiotropic effect test was used to determine whether plasma lipid-related genetic IVs could lead to FD via other potential pathways. There is no significant horizontal pleiotropy in our MR analyses (all p values > 0.05). This indicates that our MR studies were probably not affected by potential confounding pathways, and the results were robust, credible, and reliable (Table 5).

**Table 5:** The pleiotropic test of plasma lipids genetic variants in IBS and FD genome-wide summary association study (GWAS) datasets.

Traits (outcome)	Plasma lipids (exposure)	Egger intercept	SE	p
IBS	LDL-cholesterol	− 0.006	0.003	0.088
	HDL-cholesterol	0.003	0.002	0.232
	Total cholesterol	− 0.007	0.005	0.119
	Triglycerides	0.001	0.002	0.835
	Apolipoprotein A1	− 0.001	0.003	0.849
	Apolipoprotein B	− 0.003	0.003	0.366
FD	LDL-cholesterol	− 0.002	0.003	0.562
	HDL-cholesterol	− 0.143 × 10^–3^	0.002	0.952
	Total cholesterol	− 0.003	0.005	0.529
	Triglycerides	0.001	0.003	0.583
	Apolipoprotein A1	− 0.002	0.003	0.377
	Apolipoprotein B	0.001	0.003	0.706

*SE* standard error.

p < 0.05 is set as the significant threshold.

## Discussion

Till date, several metabolomic analyses have identified that plasma lipid levels, including triglyceride, fatty acid, cholesterol, and several other lipid metabolite levels, differ between patients with FGID and controls, and this variability also occurs in different types of FGID^8,11,23,24^, however, it is difficult to explain the exact causal relationship between the diseases and the change in plasma lipid levels because of the limitations of the traditional observational study methods. In contrast, MR is used to study the causal relationship between exposure and outcome using genetic variation that is randomly distributed and not influenced by confounding factors such as IVs^25^. This enables us to assess the relationship between increased plasma lipid levels and FGID. This two-sample MR study showed that increased triglyceride levels increase the risk of IBS rather than that of FD. However, there is not any causal relationship between other plasma lipids (LDL-cholesterol, HDL-cholesterol, total cholesterol, apolipoprotein A1, and apolipoprotein B) and FGID.

Plasma lipids include free fatty acids, triglycerides, and cholesteryl esters. Triglycerides are the major form of intracellular and plasma fatty acids storage and transport, which stores most of the energy for regulating physiological functions^26^. However, abnormal levels of triglyceride lead to several kinds of diseases, including coronary heart disease, acute pancreatitis, metabolic syndrome, and microvascular complications in patients with diabetes such as diabetic nephropathy and other diseases^10,27–29^.

After eliminating potential confounders such as sex, age, and the first 10 genetic principle components, we found that 312 genetic IVs acquired from the UK biobank were tightly associated with triglycerides. The maximum likelihood estimation, penalized weighted median estimator, weighted median estimator, and MR-Egger, also proofed that increased triglyceride levels resulting from genetic factors may increase the risk of IBS. The pleiotropic analysis did not show significant pleiotropic effect between triglycerides genetic variants and IBS (Table 2), and the leave-one-out analysis detected there is not a statistically significant single SNP associated with the results (Fig. 3). Based on the results, we found that plasma lipids, particularly triglycerides, affected the pathogenesis of IBS. Moreover, triglycerides showed an exact causal relationship with IBS, and no other causal relationships were detected in the remaining groups, including LDL-, HDL-cholesterol, total cholesterol, apolipoprotein A, and apolipoprotein B. The correctness and reliability of the relationship were proven by sensitivity analyses, such as the pleiotropic test. Supplementary MR methods also validated these results. Among the 12-MR analysis groups, the HDL-cholesterol groups showed significant statistical heterogeneity. We cannot determine the exact source of the heterogeneity because of the limited access to the original data; however, we speculate that it may be caused by factors such as depression, anxiety, and education. A multiplicative random-effects IVW model was performed to alleviate this effect. Further, this investigation was carried out to explore the causal effects of plasma lipids on FGID. However, traditional epidemiological studies have a difficult problem as the value of plasma lipid levels cannot be accurately detected and the intake value cannot be monitored. This could be solved by using MR. In a traditional epidemiological study, plasma lipid levels can be affected by diet, exercise, body conditions, and other factors that change the relationship with IBS. The influence of diet or other habits on plasma lipid levels could not be judged without an original article on plasma lipid GWAS statistics. But, our study explored variations in the FGID risk based on plasma lipid levels determined by genetic variants. We designed a MR analysis to avoid traditional confounders like diet and other habits by introducing IVs and monitoring their interference using sensitivity analysis. Therefore, the influence of confounding factors did not affect the results.

Based on the present study, the potential mechanisms suggesting that triglycerides are associated with an increased risk of IBS remain unknown. Therefore, we propose several potential possibilities based on the mechanisms contributing to IBS such as disturbances in the intestinal microbiota, immune activation, low-grade mucosal inflammation, and altered intestinal permeability^5^, in addition to notable influences from obesity and diet^30^. First, a previous study showed that high levels of triglyceride lead to triglyceride-rich lipoproteins enriched with apoC-III, which affects the signaling pathways that activate NFKβ and increase inflammatory molecules^31^, which suggesting that it could increase intestinal mucosal inflammation. Second, researchers suggest that the environment for microbial survival in the gut is altered by hyperlipidemia, leading to intestinal flora dysbiosis that aggravates a lipid metabolism disorder, which is an important risk factor for IBS^32,33^. Third, zonulin is considered a serum biomarker that reflects intestinal permeability, which is a manifestation of IBS^34,35^. However, the zonulin expression level in the IBS group was not different from that in the control group after adjusting for confounders. Zonulin levels are related to glucose levels, dyslipidemia, and insulin resistance^36^. Therefore, the increase in intestinal permeability could be caused by dyslipidemia, which increases zonulin levels and induces IBS. Fourth, diet has long been linked with IBS as a double-edged sword, it is a cause of diseases but also a treatment for symptom relief^37^. Some study suggested that gastrointestinal symptoms were frequently reported after intake of high-fat foods^38–40^, which can increase the level of plasma triglyceride^41,42^. According to these results, we hypothesized that there may be an increased triglyceride component to the exacerbation of IBS symptoms caused by high-fat foods. Last but not least, a systematic review suggested that obesity is also a relative risk factors of IBS^43^ and we all known that IBS patients weigh more than healthy controls, and some researches also proved that the obesity patients always have elevated plasma triglyceride levels^44^. Combined with our results it is easy to find that the mechanisms of obesity aggravating IBS may include the high-level of triglyride. In summary, the possible mechanisms of triglyceride-induced IBS are still unclear; here, we only present speculations and hypotheses based on existing studies and our results, which suggest that increased triglycerides increase the risk of IBS rather than that of FD. Our MR study had several strengths. First, we analyzed the causal relationship between plasma lipid concentrations and FGID, and this could provide a clear direction for pathological studies. Second, this study was established based on the three main assumptions of the IVs, which conform to the checklist for performing MR investigations and make our conclusions reasonable and reliable^45^. Third, this study benefited from large-scale GWAS of European ancestry, which helped in preventing bias in population stratification. Finally, we used five different MR analysis methods to ensure the consistency of the causal effects.

However, this study had some limitations. First, the outcomes cannot be generalized for all species, and the relationship may change in individuals of other ancestries. The results of the exposure (different plasma lipids) and outcome (IBS and FD) statistics for the genetic IVs were all obtained from the GWAS of European ancestry. Second, the diagnostic methods for IBS and FD could differ between hospitals and health-care systems as they follow a diagnosis of exclusion; similarly, different information acquisition and data processing methods may bias the results. Third, we did not include all dimensions of different plasma lipids in our study, as mentioned previously, because of the lack of an explored GWAS. Finally, although we removed the confounding effects of linkage disequilibrium and pleiotropy, biological mechanisms and genetic co-inheritance, such as gene expression, gene–gene interactions, and gene–environment interactions, could still affect the accuracy of our results.

In conclusion, our study provides genetic evidence that triglycerides are an important risk factor for IBS rather than FD, and this might help in the prevention of IBS if a larger, randomized, controlled cohort study is conducted. Moreover, LDL-cholesterol, HDL-cholesterol, total cholesterol, apolipoprotein A1, and apolipoprotein B levels were not associated with the risk of IBS or FD. However, our study was based on plasma lipid levels determined by genetic variants, which could only account for a part of the IBS risk variation. Therefore, a large-sample, randomized, controlled cohort study is required to clarify the association between plasma lipid levels and IBS. More types of plasma lipids should be explored to determine their causal effects on FGID through further mining and improvement of databases.

## Conclusion

Our MR study showed that triglycerides had a positive causal effect on IBS rather than on FD. LDL-cholesterol, HDL-cholesterol, total cholesterol, apolipoprotein A1, and apolipoprotein B levels were not associated with the risk of IBS or FD.

### Supplementary Information

Supplementary Figures.

## Data Availability

Publicly available datasets were analyzed in this study. The dataset(s) supporting the conclusions of this article are available in the UK Biobank [https://www.ukbiobank.ac.uk/] and FinnGen Study [https://www.finngen.fi/en/].

## Abbreviations

FGID: Functional gastrointestinal disease
IBS: Irritable bowel syndrome
FD: Functional dyspepsia
MR: Mendelian randomization
IVs: Instrumental variables
GWAS: Genome-wide association studies
UKB: UK Biobank
SNP: Single nucleotide polymorphism
IVW: Inverse-variance weighted
