# Supplementary material for: A Mendelian randomization study on the effects of plasma lipids on irritable bowel syndrome and functional dyspepsia
Source: Sci Rep. 2024 Jan 2;14:78. doi: 10.1038/s41598-023-50459-9 (PMC10761668; doi:10.1038/s41598-023-50459-9)
Supplement: Supplementary file 1 — Supplementary Figures. [file 41598_2023_50459_MOESM1_ESM.pdf]

Supplementary Material

A Mendelian Randomization Study on the Effects of Plasma Lipids on Irritable Bowel Syndrome and Functional Dyspepsia

Mengmeng Xu[1,2], Deliang Liu[1,2], Yuyong Tan[1,2], Jian He[3], Bingyi Zhou[1,2]*

Correspondence: Bingyi Zhou: zhoubingyi0508@csu.edu.cn

Supplementary Figures

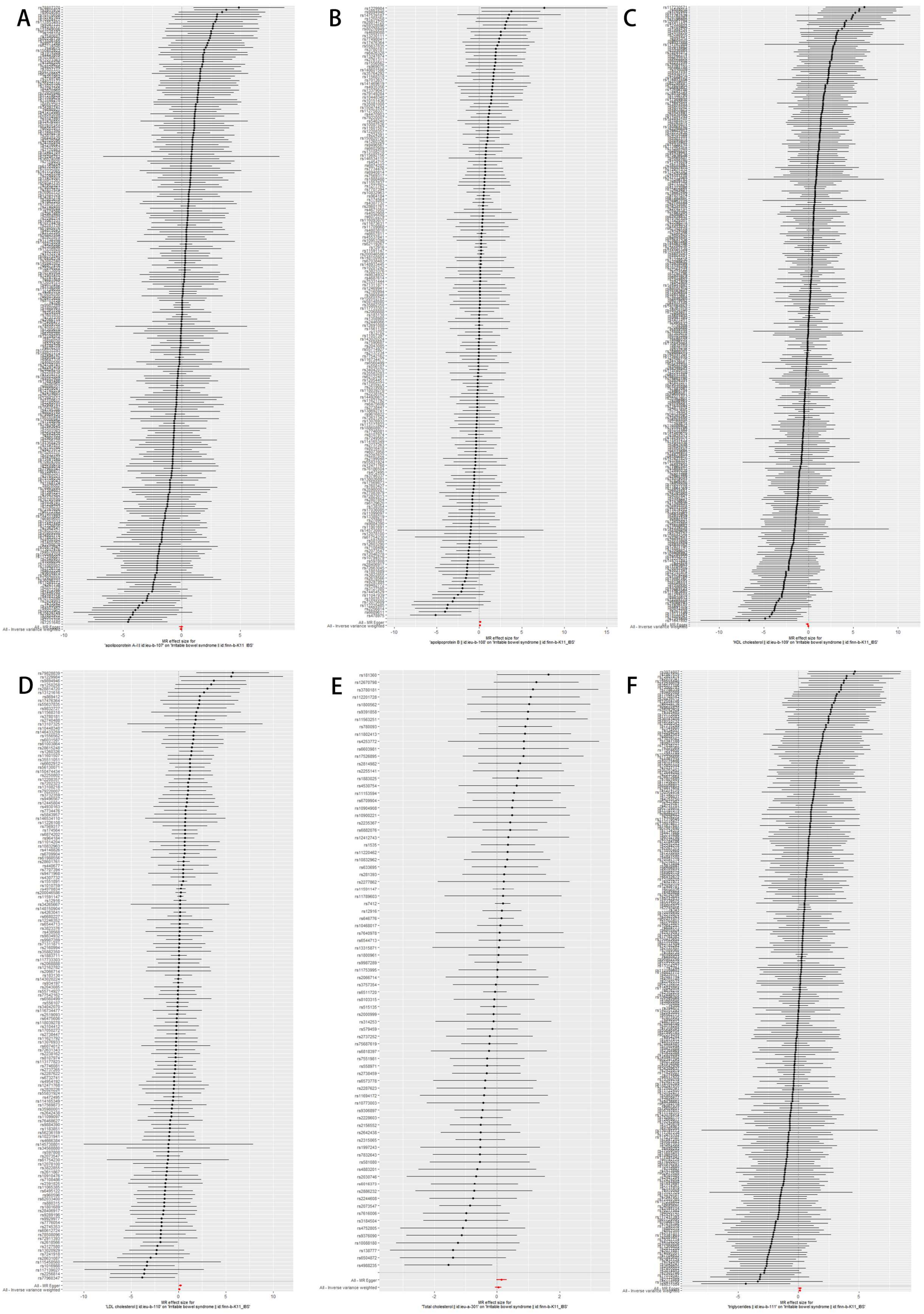

Supplementary Figure 1: Scatter plot of plasma lipids and irritable bowel syndrome (IBS). (A) apolipoprotein A1-IBS. (B) apolipoprotein B-IBS. (C) HDL cholesterol-IBS. (D) LDL cholesterol-IBS. (E) total cholesterol-IBS. (F) triglycerides-IBS. Analyses were conducted using the conventional IVW, MBE, WMM, MR-Egger, and MR.RAPS methods.

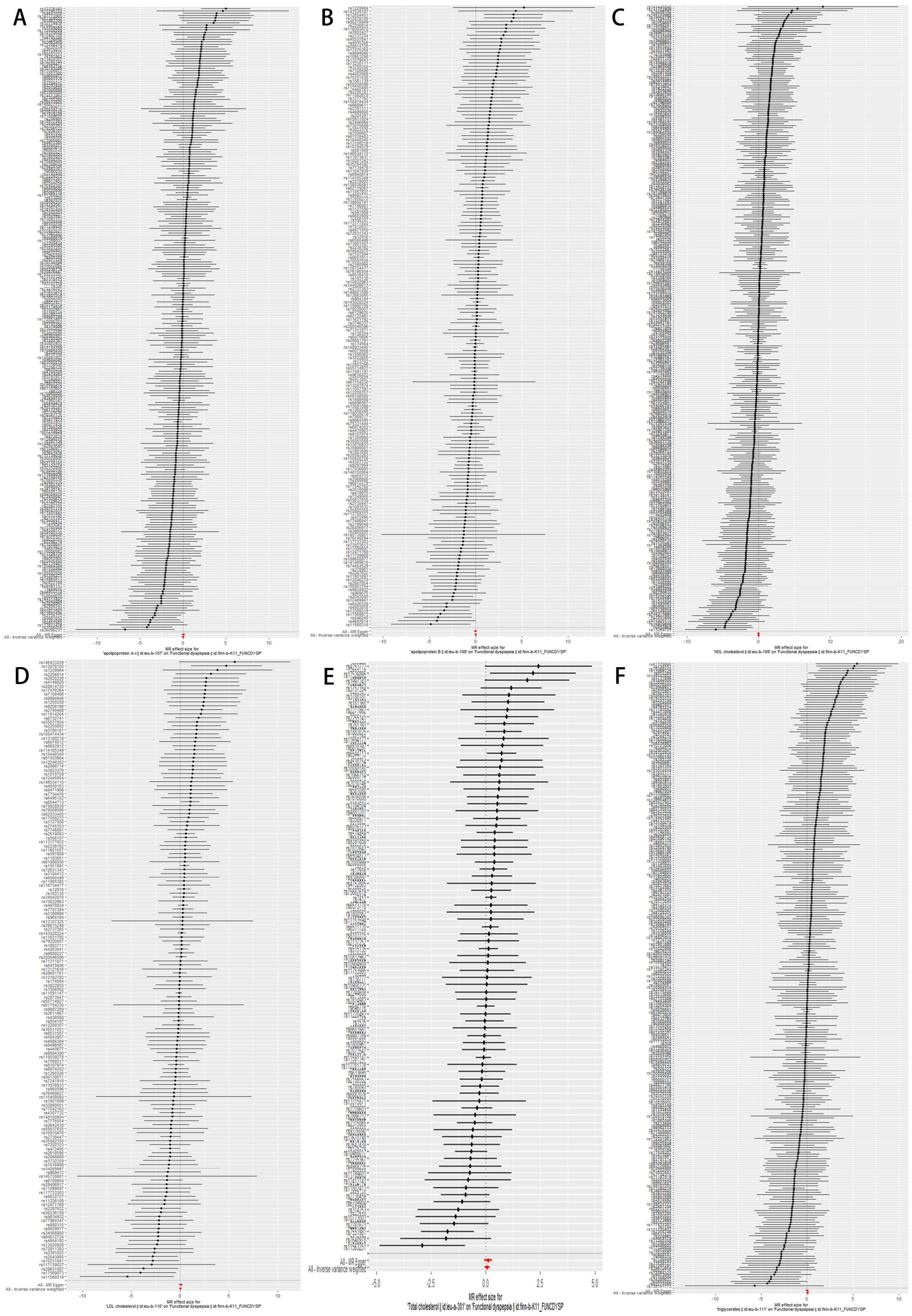

Supplementary Figure 2: Scatter plot of plasma lipids and functional dyspepsia (FD). (A) apolipoprotein A1-FD. (B) apolipoprotein B-FD. (C) HDL cholesterol-FD. (D) LDL cholesterol-FD. (E) total cholesterol-FD. (F) triglycerides-FD. Analyses were conducted using the conventional IVW, MBE, WMM, MR-Egger, and MR.RAPS methods.

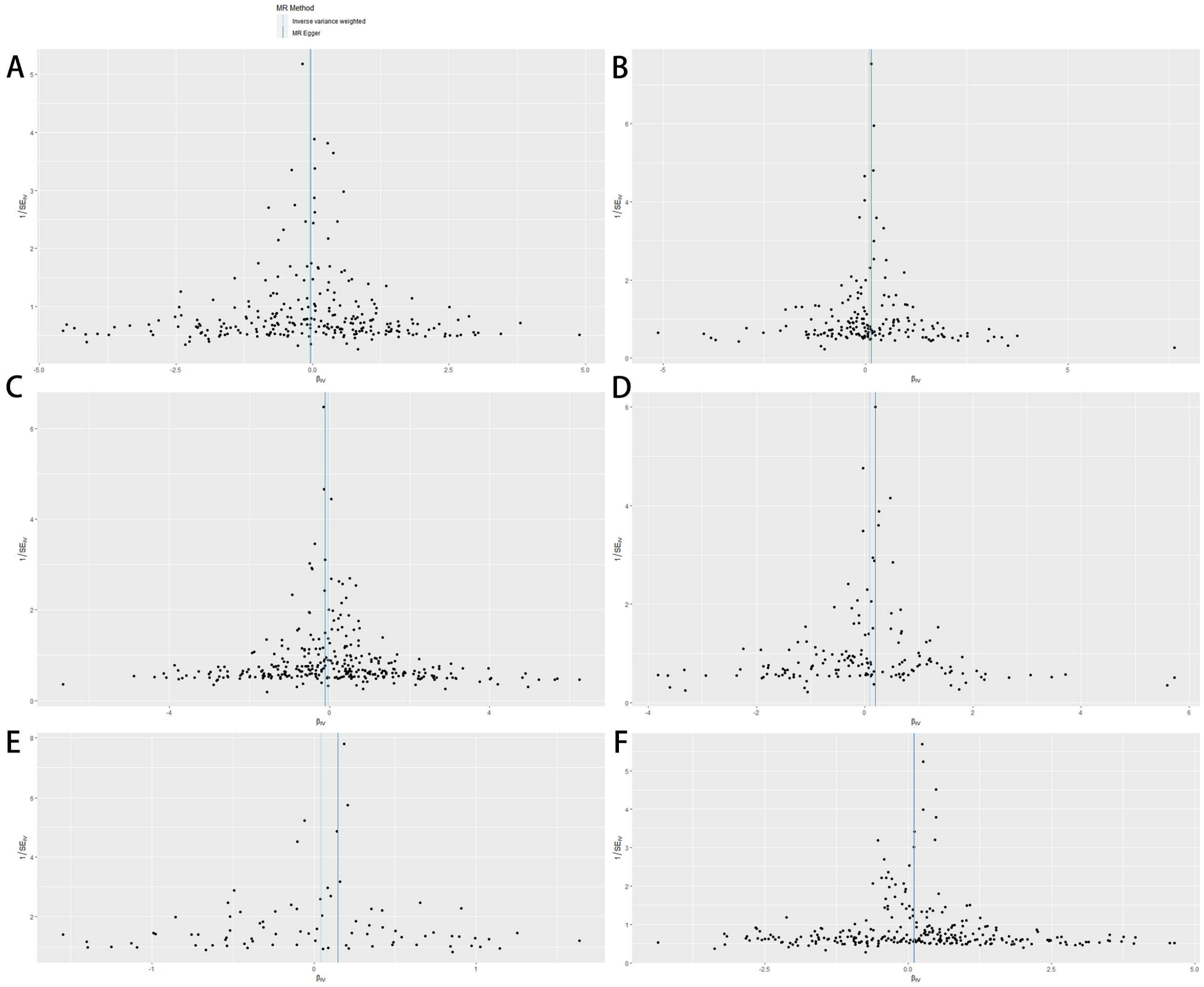

Supplementary Figure 3: Funnel plot of the inverse variance weighted MR estimate of each plasma lipid SNP with IBS versus 1/SEIV; (A) Analysis of apolipoprotein A1 and IBS; (B) Analysis of apolipoprotein B and IBS; (C) Analysis of HDL cholesterol and IBS; (D) Analysis of LDL cholesterol and IBS; (E) Analysis of total cholesterol and IBS; (F) Analysis of triglycerides and IBS.

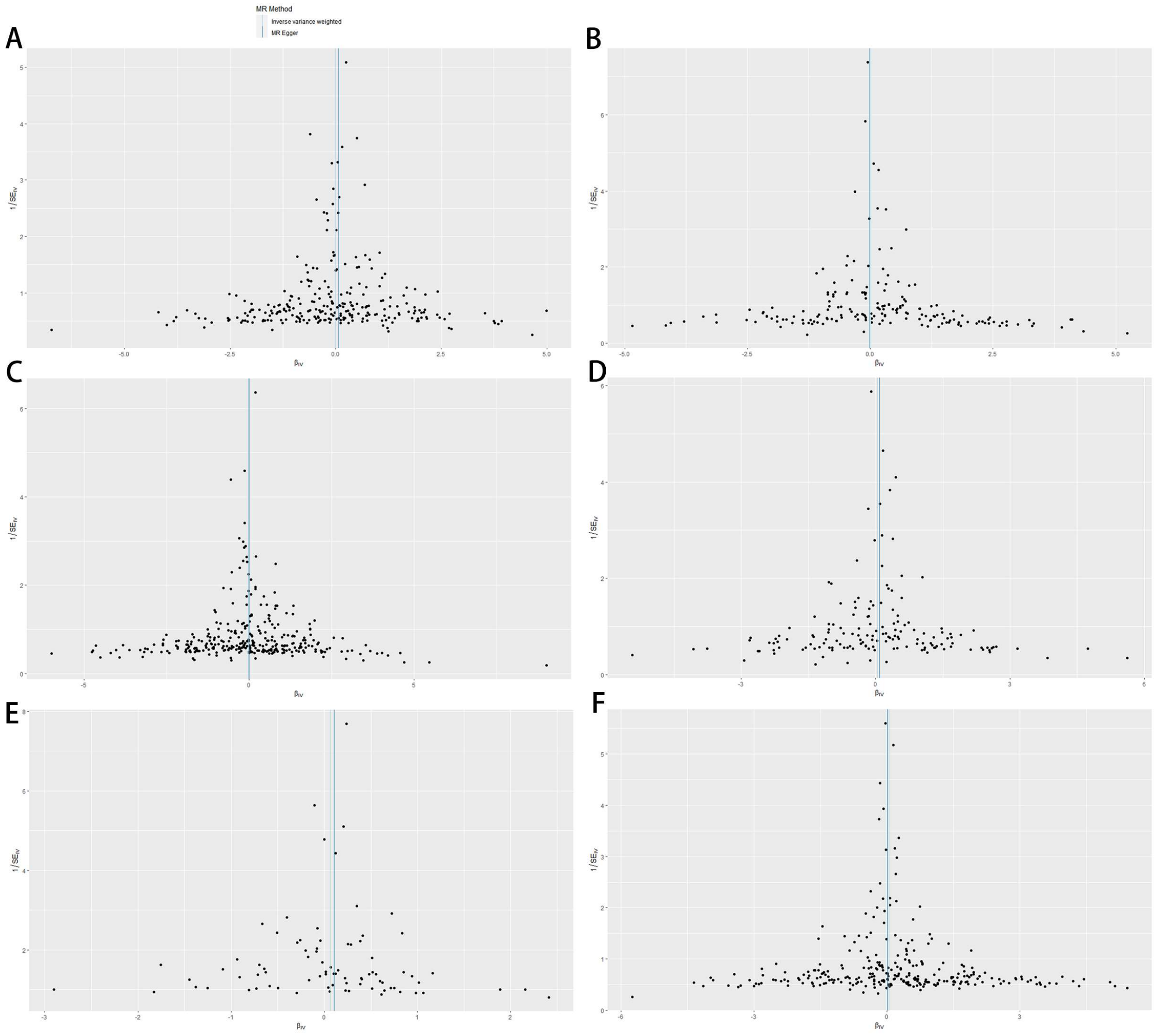

Supplementary Figure 4: Funnel plot of the inverse variance weighted MR estimate of each plasma lipid SNP with FD versus 1/SEIV; (A) Analysis of apolipoprotein A1 and FD; (B) Analysis of apolipoprotein B and FD; (C) Analysis of HDL cholesterol and FD; (D) Analysis of LDL cholesterol and FD; (E) Analysis of total cholesterol and FD; (F) Analysis of triglycerides and FD.

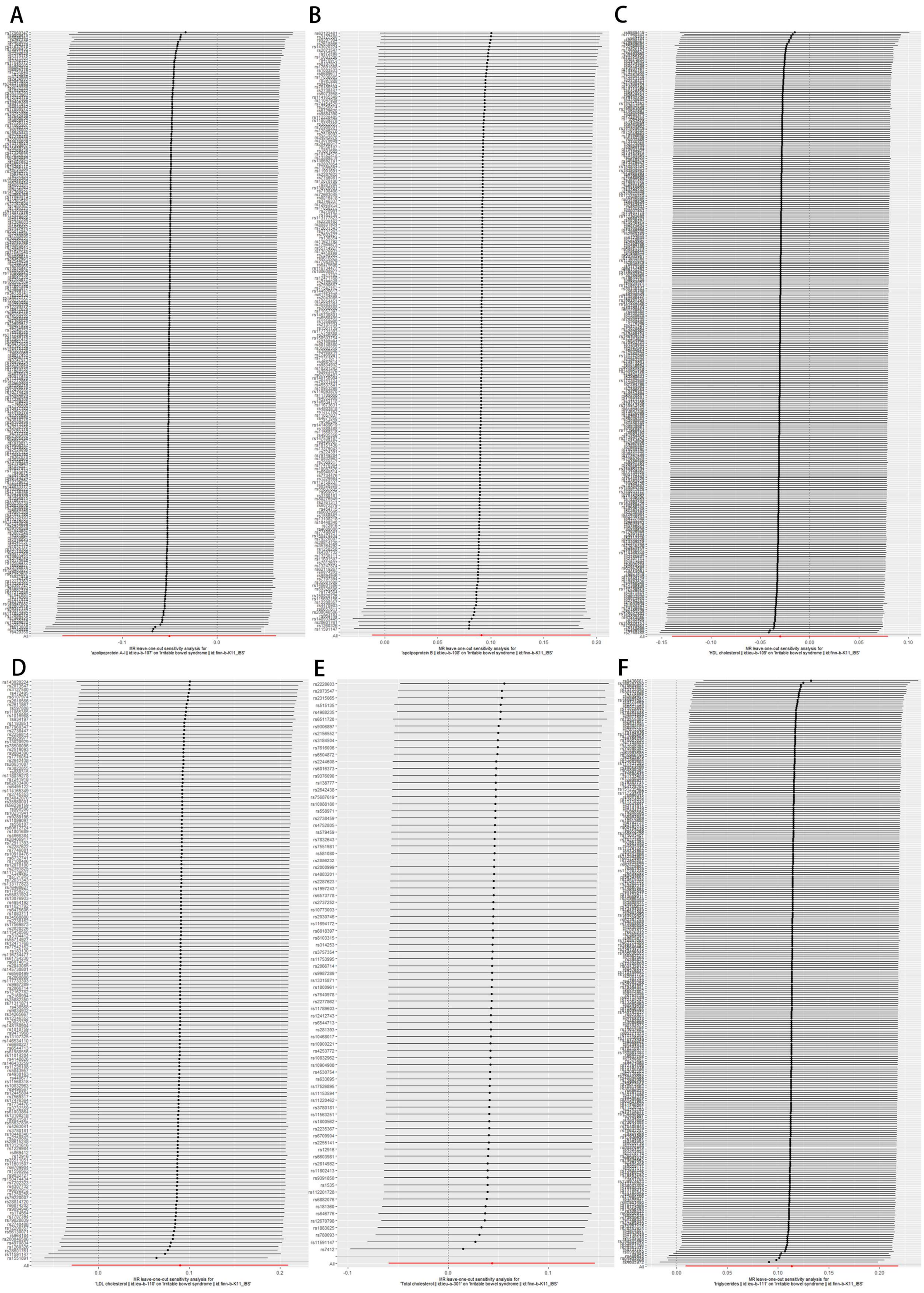

Supplementary Figure 5: Leave-one-out analysis for the effect of plasma lipid and IBS: (A) apolipoprotein A1-IBS. (B) apolipoprotein B-IBS. (C) HDL cholesterol-IBS. (D) LDL cholesterol-IBS. (E) total cholesterol-IBS. (F) triglycerides-IBS.

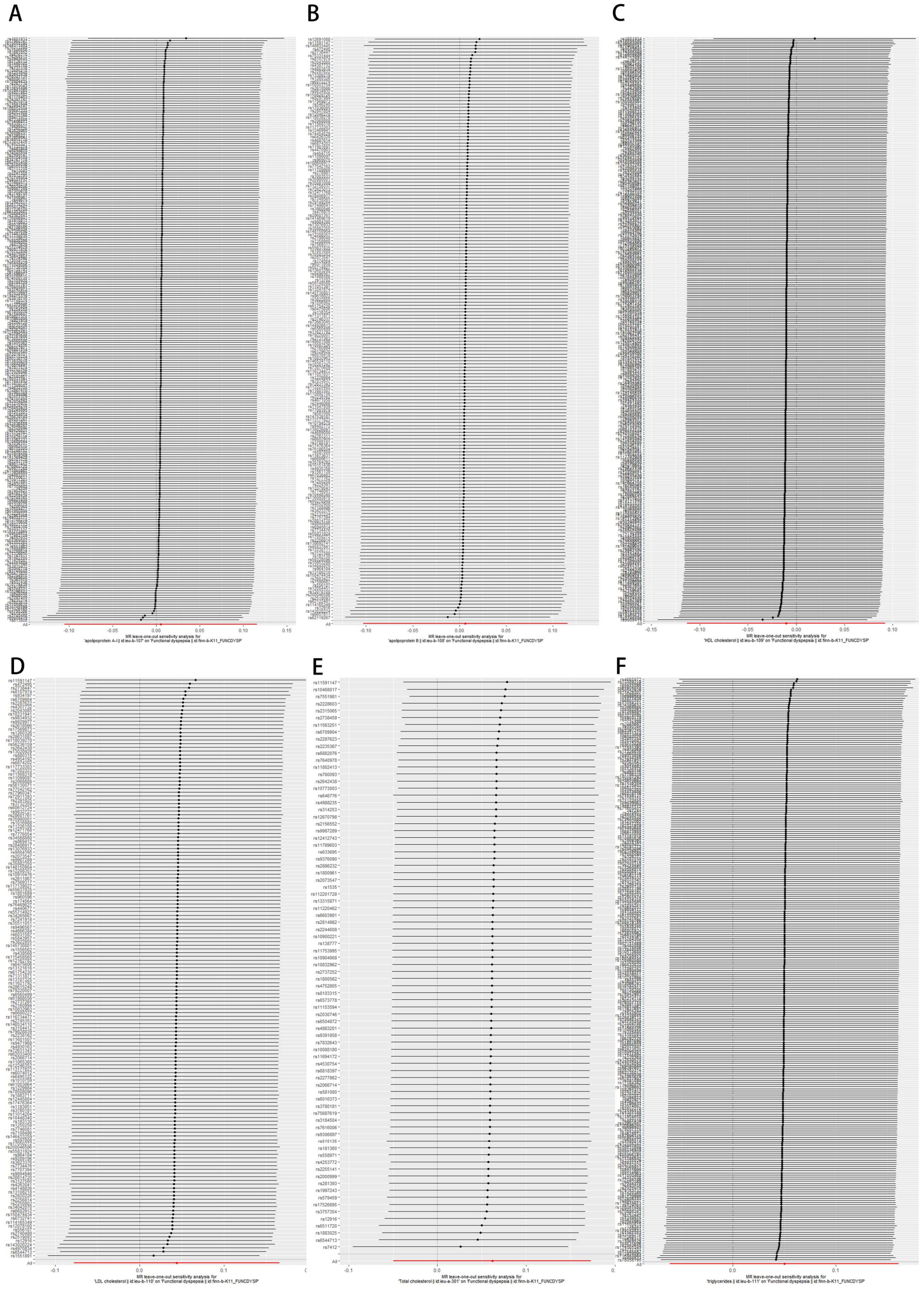

Supplementary Figure 6: Leave-one-out analysis for the effect of lifestyle factors and FD. (A) apolipoprotein A1-FD. (B) apolipoprotein B-FD. (C) HDL cholesterol-FD. (D) LDL cholesterol-FD. (E) total cholesterol-FD. (F) triglycerides-FD.
